# No Reported Renal Stones with Intravenous Vitamin C Administration: A Prospective Case Series Study

**DOI:** 10.3390/antiox7050068

**Published:** 2018-05-21

**Authors:** Melissa Prier, Anitra C. Carr, Nicola Baillie

**Affiliations:** 1 Feedback Research Ltd., Auckland 1050, New Zealand; melissa@feedbackresearch.co.nz; 2 Department of Pathology and Biomedical Science, University of Otago, Christchurch 8011, New Zealand; anitra.carr@otago.ac.nz; 3 Integrated Health Options Ltd., Auckland 1050, New Zealand

**Keywords:** vitamin C, intravenous vitamin C, renal stones, kidney stones, oxalate, renal function, creatinine, glomerular filtration rate

## Abstract

A few cases associating high dose intravenous vitamin C (IVC) administration with renal stone formation have been reported in the literature, however, no long-term studies investigating IVC administration and reported renal stones have been carried out. Our aim was to measure the frequency of reported renal stones in patients receiving IVC therapy. We carried out a prospective case series study of 157 adult patients who commenced IVC therapy at Integrated Health Options clinic between 1 September 2011 and 31 August 2012, with follow-up for 12 months. Inquiries into the occurrence of renal stones were conducted at enrolment, 6 and 12 months, and renal function blood tests were conducted at enrolment, 4 weeks and every 12 weeks thereafter in a subgroup of patients. No renal stones were reported by any patients in the study, despite 8% of the patients having a history of renal stones. In addition, the majority of patients investigated had stable renal function during the study period as evidenced by little change in serum creatinine levels and estimated glomerular filtration rate (eGFR) following IVC. In conclusion, IVC therapy was not associated with patient-reported renal stones. Although not the primary focus of this study, it was also observed that there was no significant change in mean serum creatinine or eGFR for those who had follow-up renal function blood tests.

## 1. Introduction

Intravenous vitamin C (IVC) has been used for many decades by health care practitioners for a number of indications, particularly cancer and infection [1,2,3]. IVC has been shown to be effective at decreasing common cancer-related symptoms and chemotherapy side-effects, thus improving overall patient quality of life [4]. Furthermore, recent studies indicate that IVC can improve the outcomes of patients with severe infections and burns, and improve post-surgical recovery [5,6]. Pharmacokinetic studies have found that the peak concentration of vitamin C achievable in blood plasma following oral ingestion is about 220 μmol/L, whereas at least 15 mmol/L is achievable following IVC administration [7]. One of the proposed mechanisms of IVC is to target cancer cells via an indirect pro-oxidant mechanism relying on transition metal ion-dependent generation of hydrogen peroxide; vitamin C must be injected intravenously in order to achieve sufficiently high plasma vitamin C concentrations to facilitate this mechanism [2,8]. Intravenous administration may also be required to enhance diffusion of vitamin C into solid tumours with subsequent modulation of important cell signaling pathways [9,10]. Lower IVC doses tend to be used for patients with severe infection with the proposed mechanism of IVC being primarily support of organ and immune function [5,11].

IVC is generally considered to be safe with few adverse effects, however, it has been recommended that IVC be used with caution in patients with renal impairment or failure, or a history of renal oxalate stones [1,3]. Renal dysfunction impairs the kidney’s ability to clear high doses of vitamin C from the circulation and several cases of IVC toxicity have been reported in patients with underlying renal dysfunction [12,13,14,15]. However, because haemodialysis and haemofiltration are able to remove vitamin C from the circulation [16], renal dysfunction is not necessarily contraindicated for IVC therapy in critically ill patients. In fact, there is some evidence that IVC may improve acute kidney injury in critically ill patients and also decrease renal toxicity in oncology patients receiving chemotherapy [17,18,19].

A minor product of vitamin C metabolism is oxalate and elevated urinary oxalate excretion has been reported after oral vitamin C intakes of 2 g/d, raising concerns associating vitamin C with increased risk of renal stones, which can potentially result in oxalate nephropathy [20,21]. Interestingly, neither of these studies reported a difference in vitamin C-associated oxalate generation between stone formers and non-stone formers. Robitaille et al. monitored oxalic acid secretion after IVC administration (doses of 0.2–1.5 g/kg body weight) and showed that less than 0.5% of very large IVC doses are excreted as oxalic acid in people with normal renal function [22]. Nevertheless, several cases of renal stone formation have been reported by practitioners administering IVC, although the type of stone was not always specified [1]. A clinical study in patients receiving continuous IVC for cancer also reported one case of a renal stone three weeks into the study, although renal function blood tests remained normal [23]. 

Published clinical studies on IVC administration typically only monitor outcomes for up to eight weeks, and the focus has not been on renal stone incidence or specifically renal function. There is a gap in the literature around assessment of the incidence of renal stones in patients receiving IVC over a longer time frame. There is minimal information on the time frame over which renal stones develop. Renal stones causing renal colic can develop in three months or less in high risk environments [24], although some may take longer than 12 months to develop [25,26]. Therefore, in the interests of patient safety, we sought to investigate whether IVC therapy was associated with renal stone presentation. The key parameters under investigation in this study were the patient-reported incidence of renal stones, considered reliable because of the inability to ignore renal colic which causes acute severe pain. Changes in serum creatinine and estimated glomerular filtration rate (eGFR) were also monitored in a subgroup of patients.

## 2. Materials and Methods

### 2.1. Study Design, Setting and Participants

This study was a prospective case series study which took place in a single private medical clinic, Integrated Health Options Ltd., in Auckland, New Zealand. The study received ethical approval from the Northern X Regional Ethics Committee (Reference NTX11/EXP/189).

Consecutive new adult patients seeking IVC therapy at the clinic were enrolled in the study between 1 September 2011 and 31 August 2012. To be included in the study of renal stone reporting, eligible participants were those: who had their first admission to the clinic during the study enrolment period, and received at least one IVC infusion, exclusively at the clinic, and had IVC as their main IV therapy. Exclusion criteria were patients receiving IV chelation therapy, as this may be a possible confounding factor. There were no other exclusion criteria as IVC is seen to be generally safe [1,27]. Patients were also closely monitored throughout the treatment to ensure safety (see follow-up section).

### 2.2. Study Size

The study size was determined by the number of patients who met eligibility criteria during the 12-month study period and who consented to be enrolled. Of the 168 patients who met the eligibility criteria for the renal stone reporting group, 157 were enrolled (93%). Reasons for non-enrolment could not be identified. Power calculations indicated that 125 patients would be required to achieve 80% power to detect significant changes in renal function.

### 2.3. Baseline Data

At consultation, prospective patients for whom IVC was indicated were asked if they had a history of renal stones. If the patients wished to proceed with IVC therapy, they were given a standard laboratory form and requested to visit their local phlebotomy lab for tests including glucose-6-phosphate dehydrogenase (G6PD) activity and renal function. Participant characteristics (including age, gender, history of renal stones, mean number and dose of IVC treatments, mean creatinine and eGFR), and their presenting conditions were recorded.

### 2.4. Treatment

IVC therapy involved diluting vitamin C (500 mg/mL Ascor L 500, McGuff Pharmaceuticals, Santa Ana, CA, USA) in 250–1000 mL sterile water depending on dose to maintain an appropriate osmolarity [3]. Doses started at 15–25 g per infusion and, depending on the medical condition, increased gradually over several infusions to typically between 50 and 100 g per infusion based on body-weight, plasma vitamin C concentration and patient tolerance. Two to three infusions per week plus daily oral vitamin C (1000–2000 mg/day) were recommended to maintain high plasma vitamin C levels.

### 2.5. Follow-Up

Renal function blood tests were routinely ordered for all patients prior to commencing IVC therapy and repeated after 4 weeks then every 12 weeks for as long as the patient continued to have IVC treatments at the clinic. Changes in renal function were managed in such a way that if the eGFR reduced 15–20%, renal function blood tests were repeated within 1–2 weeks, and if the eGFR reduced more than 20%, IVC was promptly ceased, the patient reviewed and any possible causes addressed [28,29]. Renal function tests were repeated 1–2 weeks later and if normalized or improving, IVC was restarted and monitored closely until stabilized. If levels continued to reduce, IVC was not restarted. 

Enrolled patients were contacted by phone 6 months and 12 months after admission and asked a standardised question about experiencing, or seeking medical advice for, symptoms of renal stones or renal colic since starting IVC. If a patient died during the follow-up period, a coroner’s report or death certificate was requested to check whether renal stones or renal impairment/failure was the cause of death or the antecedent cause of death. Due to a significant proportion of patients presenting with advanced cancer, a number of patients died during the study period (37/157, or 24%). Some patients were lost to follow-up due to moving overseas or lost to contact (three at six months, and six at 12 months). 

### 2.6. Data Sources and Measurement

The patient-reported incidence of renal stones at the initial consult and follow-up phone interviews were recorded in a spreadsheet by a doctor. For those patients who died during the study period, information recorded on their death certificates was obtained from the Deputy Registrar General of Births, Deaths and Marriages. Other data collected by the treating physician included patient demographics, number of IVC infusions, renal function laboratory results and comments on reason for treatment cessation. Telephone interviews were conducted by the clinic nurses to investigate reported renal stones even after the patient stopped treatment. The last recorded renal function blood tests were used for comparison with their pre-IVC tests to measure changes in renal function.

### 2.7. Statistical Analysis

Statistical analyses were performed in Microsoft Excel 2007 (Microsoft Corporation, Redmond, WA, USA) with the Real Statistics 2.16.2 (Charles Zaiontz, http://www.real-statistics.com) data analysis tools add-on to conduct non-parametric tests. The Wilcoxon Signed-Rank test was used for paired samples (i.e., before and after), the Mann–Whitney test was used to compare two independent groups and the chi-squared test was used to compare groups with qualitative variables such as gender. A two-tailed *p*-value less than 0.05 was considered statistically significant. 

## 3. Results

### 3.1. Participants

Of 168 new patients presenting to the clinic during the 12-month enrolment period, 157 (93%) eligible patients were enrolled in the study. At six months 154 (98%), and at 12 months 148 (94%) were able to be followed up by phone interviews, or death certificate information regarding reported renal stones. Approximately half (78/157) of the patients stopped IVC therapy before follow-up renal function blood tests were completed (i.e., within four weeks). Patient characteristics are presented in Table 1; it should be noted that 12 (8%) of the patients had a history of renal stones. The major presenting conditions were cancer and infectious diseases or requiring immune support (Table 2).

### 3.2. Outcome Data

#### 3.2.1. Reported Renal Stones

**Figure 1:**
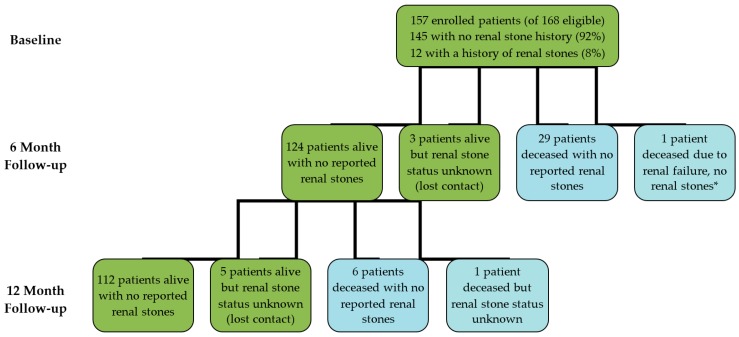
Flow diagram of participants involved in analysis of reported renal stones. * See Patient A case report.

Six months after commencing IVC therapy, 153 out of 157 patients (124 alive patients and 29 deceased patients) had no reported renal stones or no renal stones causing renal impairment/failure recorded on death certificates (Figure 1). One patient with pre-existing renal problems had died of renal failure (refer to Patient A case report below) and three patients had moved overseas and lost contact, so their renal stone status could not be determined. At 12 months, 118 (112 alive patients and six deceased patients) had no reported renal stones or no renal stones causing renal impairment/failure recorded on death certificates (Figure 1). Contact was lost with six patients (four patients had moved overseas, one was deceased, and failed to make contact with one), so their renal stone status could not be determined.

#### 3.2.2. Renal function tests

Paired data for renal function blood tests included 79 and 71 patient results for creatinine and eGFR, respectively. Forty-five (57%) of the patients showed an increase in serum creatinine concentrations, although only 3 of these went above normal (Table 3). The mean change in serum creatinine concentration was +1.8 ± 11.0 µmol/L (4.1 ± 15.7%). This was not statistically significant and was within reported biological variation expectations (4.7% in healthy individuals and 8.9% in people with renal impairment) [30]. Only 6 (8%) of the patients showed a decrease of ≥15% in eGFR (Table 3). The mean change in eGFR was −0.7 ± 9.9 mL/min/1.73 m^2^ (0.1 ± 13.5%) and was not statistically significant. There was no significant relationship between gender (chi-squared *p*-value > 0.05).

### 3.3. Patient A Case Report

Patient A died due to renal failure within six months of starting the study, but there were no reported renal stones, nor did she have a history of renal stones. The patient was a 60-year-old woman with metastatic breast cancer diagnosed four and a half years earlier who had undergone a mastectomy, axillary dissection, chemotherapy and hormone treatments. She had been admitted to hospital for pulmonary emboli, post-renal obstruction from ascites, peritoneal spread and gastric invasion five weeks prior to presenting at the clinic. Her renal function had improved following drainage of ascites. Four weeks prior to attending the clinic, she had started a course of chemotherapy with vinorelbine, had two treatments then two weeks off and further treatment was discontinued due to increased liver function test results.

After her consultation at the clinic she had three IVC treatments in the week following. She was readmitted to hospital 11 days after starting IVC with recurrence of post-renal obstruction and ascites. Renal function did not improve despite further drainage of ascites. She died four days later.

The possibility that IVC contributed to Patient A’s renal failure cannot be excluded, but it is considered unlikely as she had proven recurrence of a pre-existing condition that had recently caused acute renal injury, namely post-renal obstruction secondary to cancer-related ascites. There were no renal stones reported from the investigations performed in hospital, or on her death certificate.

## 4. Discussion

The primary outcome of this prospective study was that no renal stones were reported by any patient who received IVC during the study period, despite 8% of participants reporting previous episodes of renal stones. This study showed that IVC was not associated with any cases of symptomatic renal stones or renal stones causing renal failure/death in 100% of patients able to be followed up over a 12-month period (94% follow-up). Although not the focus of this study, half of the eligible patients had follow-up creatinine and eGFR results available. Of the patients that provided both before and after renal function blood tests (50% of the patients), IVC therapy was not associated with a significant detrimental effect on renal function. 

The purported risk of renal stones related to vitamin C has been based mainly on increased oxalate excretion rather than actual occurrence of renal stones [20,21,31], and issues around possible ex vivo formation of oxalate during sample storage and processing have been highlighted [22,32]. Large epidemiological studies have shown no association between vitamin C intake or status and increased prevalence of renal stones, particularly in women [33,34,35,36], although several studies showed increased risk among men [36,37,38]. Epidemiological studies can, however, only indicate associations, not causal relationships and are hampered by many confounders such as fluid intake and a lack of information on renal stone composition [36,38]. Furthermore, many rely upon self-reported food frequency questionnaires which are not necessarily a good indicator of plasma vitamin C status [39]. Simon et al. measured circulating vitamin C levels and found no association with renal stones in a large cohort of over ten thousand US adults [33]. 

There have been numerous case reports in the literature of oxalate nephropathy secondary to high dose oral vitamin C intake [40,41,42,43,44,45,46,47,48,49]. However, a number of these cases are complicated by dehydration due to diarrhoea and vomiting [42,43], or acute kidney injury and transplantation requiring dialysis [46,47,48]. Although no significant changes in oxalate metabolism were observed in a study involving ten end-stage renal patients who took 100 mg/day of vitamin C [50], higher doses have been associated with increased plasma oxalate levels in haemodialysis patients [51]. However, it should be noted that oxalate excretion may be increased in haemodialysis patients even without vitamin C administration [52]. 

Nevertheless, for those with severe renal impairment or at risk of severe renal impairment, such as in the case of Patient A, the warning to be cautious with regard to administering IVC therapy should be heeded [3]. In support of this, a previous case report that linked IVC with oxalate nephropathy involved a single infusion of 45 g IVC for a patient already diagnosed with a nephrotic syndrome [13]. Another case study involved a single 60 g IVC infusion of a patient with a metastatic carcinoma of the prostate and renal insufficiency [14]. Neither of the reports showed full renal function blood tests and both patients were renally impaired prior to IVC treatment. In the clinic where the current study was conducted, renal function blood tests are frequently monitored, especially in those patients who have renal impairment, and IVC would not commence in cases of severe renal impairment or be ceased if significant progressive deterioration in renal function occurred. 

Ours is the first long-term study investigating IVC and reported renal stones. A strength of this study is that adult patients of various ages presented with a wide variety of conditions and were not excluded from the study based on their renal function prior to attending the clinic. They also received a wide range of IVC doses (from 1 g to 119 g). Therefore, the study population may be considered representative of adults receiving IVC therapy in a clinical setting. Although the incidence of renal stones in the general population is only 1–3/1000/year [53], our cohort consisted of 12 participants with a history of renal stones, which can increase the risk of developing recurrent stones by up to 50% [54,55]. Despite this increased risk and the high doses of IVC being administered, which require clearance by the kidneys, we did not observe any recurrent renal stones in these participants over the duration of the study. 

There were a number of limitations to this study. There was a potential for self-selection bias as patients could choose to stop IVC therapy or not undergo scheduled renal function blood tests. Although only 6% were lost to follow-up, 11 eligible patients were not enrolled for reasons that could not be identified. There were also confounding factors such as pre-existing disease and complications or co-morbidities, other concurrent or recent therapies and lifestyle factors such as diet. This study relied on patient-reported renal stones and for those patients who died, information was taken from their death certificates. Although self-report of renal stones has been validated [56,57], the study did not include radiographic investigations of the participants so would not have detected asymptomatic renal stones [58]. The study was also not sufficiently powered to make statistically significant conclusions regarding renal function. Furthermore, in relation to the timeframe of the study relative to renal stone development, some renal stones may take longer than 12 months to develop/cause symptoms [25,26], in which case they would not have been included in this study.

## 5. Conclusions

No renal stones were reported by patients or recorded on death certificates in 157 patients receiving IVC therapy during a 12-month period, despite 8% of the patients having a history of renal stones. The majority of patients had stable renal function and our data suggests that changes in eGFR were negligible on average, but due to statistical weaknesses (numbers lost to follow-up in this group) these findings cannot rule out the possibility that IVC therapy may contribute to renal dysfunction. Therefore, further research is required with larger sample sizes, longer term follow-up (>12 months) and radiographic investigations. 

## Figures and Tables

**Table 1:** Characteristics of participants.

Characteristic	Total Cohort ^1^	No History of Renal Stones	History of Renal Stones	*p* Value ^2^
*N* (%)	157 (100) ^3^	141 (90)	12 (8)	
Mean age, years (range)	54 (17–86)	54 (17)	58 (12)	0.332
Male/female, *n*	62/95	53/88	9/3	0.038
Mean number of IVC treatments, n (range)	15 (1–119)	14 (19)	16 (13)	0.799
Mean IVC dose per session, g (range)	50 (15–125)	49 (23)	59 (24)	0.159
Mean creatinine pre-IVC, µmol/L (SD)	70 (20)	70 (21)	76 (18)	0.323
Mean eGFR pre-IVC, mL/min/1.73 m^2^ (SD)	82 (12)	82 (14)	81 (14)	0.932

^1^ Ethnicity: European 77%, Asian 8%, Maori/Pacific people 6%, Middle Eastern 1%, not stated 8%. ^2^ Comparisons between the two subgroups, unpaired *t* test used for continuous data, and chi-square test used for categorical data. ^3^ Separate data for patients with unknown history of renal stones are not shown (*n* = 4).

**Table 2:** Presenting conditions of participants.

Presenting Condition	Total Cohort *N* (%)	No History of Renal Stones	History of Renal Stones
Cancer	76 (48)	66 (47)	6 (50)
Infectious diseases and/or immune support	54 (34)	50 (35)	4 (33)
Neurological, musculoskeletal, and skin disorders	13 (8)	11 (8)	2 (17)
Fatigue	6 (4)	6 (4)	0
Other (e.g., pre-surgery, Crohn’s disease, dental, fracture, haemorrhoids)	8 (5)	8 (6)	0

**Table 3:** Changes in participant renal function.

Observation	Patient *N* (%)
Creatinine Increased ^1^	45/79 (57)
*Low to Normal*	2/45 (4)
*Normal to Normal*	40/45 (89)
*Normal to High*	3/45 (7)
eGFR Decreased ≥ 15% ^2^	6/71 (8)
*Normal to Low*	2/6 (33)
*Low to Lower*	4/6 (66)
Creatinine Increased + eGFR Decreased ≥ 15%	6/79 (9)

^1^ Normal Creatinine: 60–105 µmol/L males, 45–90 µmol/L females. ^2^ Normal eGFR: ≥ 90 mL/min/1.73 m^2^.
